# All GLP-1 Agonists Should, Theoretically, Cure Alzheimer’s Dementia but Dulaglutide Might Be More Effective Than the Others

**DOI:** 10.3390/jcm13133729

**Published:** 2024-06-26

**Authors:** Jeffrey Fessel

**Affiliations:** Department of Medicine, University of California, 2069 Filbert Street, San Francisco, CA 94123, USA; jeffreyfessel@gmail.com; Tel.: +1-415-563-0818

**Keywords:** GLP-1 agonists, Alzheimer’s dementia, cure, brain cells

## Abstract

Addressing the dysfunctions of all brain cell types in Alzheimer’s disease (AD) should cure the dementia, an objective that might be achieved by GLP-1 agonist drugs, because receptors for GLP-1 are present in all of the main brain cell types, i.e., neurons, oligodendroglia, astroglia, microglia, endothelial cells and pericytes. This article describes the benefits provided to all of those brain cell types by GLP-1 agonist drugs. The article uses studies in humans, not rodents, to describe the effect of GLP-1 agonists upon cognition, because rodents’ brains differ from those of humans in so many ways that results from rodent studies may not be totally transferable to humans. Commercially available GLP-1 agonists have mostly shown either positive effects upon cognition or no effects. One important reason for no effects is a reduced rate of entering brain parenchyma. Dulaglutide has the greatest entry to brain, at 61.8%, among the available GLP-1 agonists, and seems to offer the best likelihood for cure of AD. Although there is only one study of cognition that used dulaglutide, it was randomized, placebo controlled, and very large; it involved 8828 participants and showed significant benefit to cognition. A clinical trial to test the hypothesis that dulaglutide may cure AD should have, as its primary outcome, a 30% greater cure rate of AD by dulaglutide than that achieved by an equipoise arm of, e.g., lithium plus memantine.

## 1. Introduction

Cure should be the goal of therapy for Alzheimer’s dementia (AD). One approach is to address all of its major, causal factors but that requires administration of an unfeasible number of drugs [1]. Since the ultimate, underlying cause of the dementia is dysfunction of brain cells, addressing all of those dysfunctions is another approach to curing it; using that approach, GLP-1 agonists may cure the dementia because, as shown below, all of the major, dysfunctional brain cell types in AD, including neurons, oligodendroglia, astrocytes, microglia, endothelial cells, and pericytes, express receptors for the glucagon-like peptide 1 (GLP-1).

The account by Holst provides a brief introduction to the 30-amino acid peptide hormone, glucagon-like peptide 1 (GLP-1) [2]. GLP-1 “is a product of the glucagon gene. The primary translation product, proglucagon, a peptide of 160 amino acids, contains apart from the glucagon sequence, two glucagon-like sequences designated GLP-1 and GLP2. They are glucagon-like because, with respect to amino acid sequence, they are about 50% homologous to glucagon. When the prohormone is processed the glucagon sequence is cleaved out, whereas the part containing the GLPs is secreted as a single, large peptide”.

## 2. GLP-1 and Brain Cells

### 2.1. GLP-1 and the Brain

GLP-1 agonists have been extensively studied in relation to their effect upon energy metabolism and nutrition. In that respect, and a link with GLP-1, a risk factor for developing AD is being underweight [3], a condition that is countered by GLP-1 agonists. In fact, nutritional status of AD patients is significantly compromised and tends to be worsened with the progression of AD [4]. Further links between a GLP-1 agonist and AD are the facts that a disturbed circadian rhythm occurs in AD [5], that blood levels of circadian clock proteins are increased in sleep apnea [6] and that the GLP-1 agonist, tirzepatide, benefits sleep apnea. GLP-1 receptors exist in various brain regions, including the nucleus accumbens [7], and the brain stem where GLP-1 activated paraventricular signaling mounts a whole-organism response to stress [8]. Data in the following sections show GLP-1 receptors in all brain cell types.

### 2.2. GLP-1 and Neurons

GLP-1 is widely present in the brain, where it is neuroprotective by reducing neuronal apoptosis, and by promoting both neurite outgrowth and synaptic plasticity [9]. The neuronal marker c-fos shows neuroanatomical connections [10], and enabled the demonstration that peripherally administered GLP-1 increased neuronal expression in the brainstem and amygdala [11]. GLP-1 receptors are abundant in the c brain stem [12] where preproglucagon neurons in the solitarius nucleus produce GLP-1 [13], and project to many regions including the hypothalamus. In the arcuate nucleus of the hypothalamus, which contains GLP-1 receptors [7], the GLP-1 agonist liraglutide caused activation of pro-opiomelanocortin neurons and inhibition of neuropeptide Y/agouti-related peptide neurons via post-synaptic GABA_A_ receptors, but enhancement of pre-synaptic GABAergic neurons [14]. GLP-1R mRNA expression was also seen in both cultured, embryonic primary cerebral cortical neurons and ventral mesencephalic (dopaminergic) neurons, both of which are vulnerable to hypoxia- and 6-hydroxydopamine-induced cell death, from which GLP-1 conferred protection [15].

As regards the effect of GLP-1 in AD, it reduced the effects of Aβ and plaque formation in AD model mice [16]; and measures of nutrition, with which GLP-1 is strongly connected, were associated with mortality in patients with AD [3,17]. That is notable because in a study of 79 patients with AD, 22 died during five years, and being underweight was a major risk factor for that mortality, with a hazard ratio (HR) of 3.34, and poor nutrition had a HR of 5.69 [3].

### 2.3. GLP-1 and Oligodendroglia

Oligodendrocytes, which carry a GLP-1 receptor [18], have a key role in the myelination of neurons, and are decreased in AD [19]. After spinal cord injury, administration of the GLP-1 agonist, exenatide, led to a significant increase in survival of oligodendrocyte progenitor cells [20], and those pre-oligodendrocytes were decreased in a mouse model of AD [21].

### 2.4. GLP-1 and Astrocytes

The presence of GLP-1 receptors in astrocytes was demonstrated by Reiner et al., who found that the uptake of a systemically administered fluorophore-tagged, GLP-1 agonist exendin-4 was blocked by pretreatment with the competitive GLP-1R antagonist exendin-(9–39) [22]. The addition of GLP-1 reduced the declines in glycolysis in astrocytes that had been induced by Aβ [23]; and liraglutide administered to AD patients prevented a decline of glucose metabolism in their brains but did not benefit cognition [24].

### 2.5. GLP-1 and Microglia

Microglia express receptors for GLP-1 [25], probably accounting for the anti-inflammatory effects of GLP-1 agonists: liraglutide caused significantly decreased levels of IFN-γ, TNF-α, and IL-6 [26]; and semaglutide led to reductions in CRP that were positively correlated with reductions in bodyweight, waist circumference, fasting plasma glucose, and fasting serum insulin [27,28].

### 2.6. GLP-1, Endothelial Cells, and Pericytes

Endothelial cells (EC) from human coronary arteries, expressed the receptor for GLP-1 [29]. EC are among those protected by the inhibition of reactive oxygen species (ROS) that is induced by GLP-1 [30,31]. GLP-1 agonism also induced up-regulation of miR-155 expression in endothelial progenitor cells [32]. The GLP-1 agonist exenatide prevented high-glucose and lipid-induced endothelial dysfunction in cultured human arterioles [33]. Pericytes were also protected by GLP-1 against the toxicity produced by ROS [34]. Pericytes have contractile properties, and control the cerebral microvascular flow (CMF) [35,36]. Because the CMF is dysfunctional in AD [37,38], its protection by GPL-1 agonists has a potential therapeutic benefit.

## 3. Other Actions of GLP-1 Agonists Relevant to AD

In addition to providing a direct benefit to brain cell types, GLP-1 receptor agonists produce an indirect benefit to them by preventing the cytotoxic effects caused by ROS [31]. Several mechanisms may account for this inhibition of ROS. First is the inhibition of NOX4, p47phox, and Rac-1 expression, and the translocation of p47phox [31]. Next is the reversal by GLP-1 of the down-regulation of histone deacetylase-6 which is produced by ROS [30]. Third is a multicomponent feedback loop that causes a stable left shift of the ROS dose–response curve; that left shift causing overproduction of ROS was prevented by GLP-1 [39].

## 4. Discussion

If the premise is correct, that addressing all of the affected brain cell types might cure AD or any other neurodegenerative disease [40,41], then this article shows that GLP-1 agonists should cure AD, because they address neurons/synapses, oligodendroglia, astroglia, microglia, endothelial cells and pericytes. However, as a class, GLP-1 agonists fall short of curing AD, so either the premise is incorrect or there is some other explanation for the failure. It is improbable that the premise is incorrect, since all neurodegenerative disease results from dysfunction, however generated, of some or all brain cell types [40]. The likeliest explanation comes from data showing that the available GPL-1 agonists have different percentages of either their entry to the brain, or in their beneficial effects.

### 4.1. Available GLP-1 Agonist Drugs

The GLP-1 agonist drugs that are currently available, mostly approved for use in the control of diabetes mellitus, are dulaglutide, exendin, liraglutide, lixisenatide, semaglutide, and tirzepatide. Exendin and lixisenatide are non-acylated and non-PEGylated and have significant blood-to-brain influx, whereas liraglutide and semaglutide do not measurably cross the blood brain barrier (BBB) [42]. However, peripheral injection of fluorescently labeled liraglutide in mice revealed the presence of the drug in the circumventricular organs, and in neurons within the arcuate nucleus (ARC) and other discrete sites in the hypothalamus [43], so that both liraglutide and semaglutide, may enter the brain by other means than via the BBB.

#### 4.1.1. Drug Presence in Brain Parenchyma versus Brain Capillaries

Because drugs exert their effects in the brain parenchyma, it is important to differentiate between their presence in parenchyma versus in the plasma within brain capillaries. For the commercially available GLP-1 agonists, the percent in the brain parenchyma versus brain capillaries was highest, at 61.8%, for dulaglutide, and their relative brain uptake as compared with dulaglutide’s was only 28% for exenatide, 14% for lixisinatide, and virtually zero for liraglutide, semaglutide, and tirzepatide [44]. Those percentages derive from the relative rates (Ki) of significant brain uptake one hour after their iv injection. In another study, the brain tissue-to-plasma partition coefficient (K_p_) of liraglutide was estimated as less than 0.00031, indicating an insignificant distribution to the brain parenchyma [45]. Transit from plasma to brain parenchyma requires crossing the BBB, which means that liraglutide, semaglutide and tirzepatide, must enter the brain at points where the BBB is minimally effective, e.g., circumventricular regions, nasal epithelium, and subarachnoid vasculature [46].

#### 4.1.2. Results from Studies in Rodents May Not Be Totally Transferable to Humans

Some of the above data are taken from studies in rodents because they could not have been obtained by studies in humans. The following data, however, that refer to the effect of GLP-1 agonists upon cognition, are taken from studies in humans, and are important because brains of rodents differ from brains of humans in so many critical ways, that the results from studies in rodents may not be totally transferable to humans. Those differences between human and rodent brains are described in detail in another article [47]. Briefly, the human astrocyte has a 27-fold greater volume than the rodent one and, therefore, benefits the human brain far more than the rodent one [48]; there is an over 2-fold greater ratio of total glia (astrocytes, oligodendroglia, and microglia) to neurons in human than rodent brain [49,50], making rodent neurons more susceptible than are human neurons to the effects of toxins such as amyloid oligomers; and 85% of the striatum in humans, forms circuits with the caudate nucleus and anterior putamen, for which there are no clear mouse homologs [51]. That absence is a difference that may lead to an interpretation that brain imaging after various treatments of rodent models had improved when, in fact, no improvement had occurred in brain areas relevant to human cognition.

### 4.2. GLP-1 Agonists and Human Cognition or Dementia

Studies regarding GLP-1 agonists affecting cognition in humans, show either positive effects or no effect, but those studies are few. In a paper published in 2023, Monney et al. found only 14 articles involving humans, that dealt with the effects of GLP-1 agonists on either cognition or AD; the agonists involved were liraglutide (in 9), exenatide (in 5), and dulaglutide and semaglutide (each in one study), and there were ongoing studies, using exenatide in 3, semaglutide in 3, and liraglutide in 3 [52]. Since it had been shown that only 1.5–2.0% of plasma GLP-1 circulates in the central nervous system [53], Monney et al., raised the question as to whether higher dosages might be needed to demonstrate benefit for cognition. Two years earlier, Norgaard et al. assessed exposure to GLP-receptor agonists in subjects with diabetes, using pooled data from15,820 patients in three randomized, double-blinded, placebo-controlled studies, and from 120,054 patients in a nation-wide, Danish, registry-based cohort [54]. Patients in the randomized studies used either liraglutide (*n* = 9340) or semaglutide (*n* = 6480) and those using the GLP-1 agonist had a mean age of 64.6 years with 24.8% aged ≥ 70 years. For those in the nation-wide cohort, each patient at the date of dementia diagnosis was matched on age, sex, and calendar date with ten controls without dementia; the GLP-agonists used in this cohort were not specified. They found that the HR for dementia was lower in both the randomized studies (HR 0.47 [CI 0.25–0.86]) and the nation-wide cohort (HR 0.89 [95% CI 0.86–0.93]).

### 4.3. Liraglutide and Cognition or Dementia

Liraglutide gave a positive effect on cognition in AD patients treated for 6 months [24], and also in a group of 16 subjects with either prediabetes or established diabetes [55]. No effects of liraglutide on cognition were found in individuals with subjective cognitive complaints, and who received liraglutide for 12 weeks [56].

### 4.4. Exenatide and Cognition or Dementia

Exenatide has also promoted variable cognitive benefit. Positive effects were seen in patients with raised intracranial pressure [57]. Administered to patients with Parkinson’s disease, cognition as reflected by the MDS-UPDRS scale, that only weakly reflects cognition, was increased in those receiving exenatide by 2.7 points and declined by 2.2 points in the control patients (*p* = 0.037) [58]. No effects or, even, worsening in females, were seen in a 32 week study [59]. Although no benefit from exenatide upon cognition was seen, it produced a reduction of Aβ42 in extracellular vesicles [60].

### 4.5. Semaglutide and Cognition or Dementia

For semaglutide, analysis of data from clinical pharmacology trials involving 376 subjects and 14,897 PK observations, showed only 0.8% bioavailabilty when it was administered with recommended dosing [61]. Although semaglutide did not cross the BBB, it could directly access the brainstem, septal nucleus, and hypothalamus via several sites surrounding and adjacent to the ventricles [62]. Exposure of a cell culture to Aβ_25–35_ inhibited autophagy, which is a feature known to affect AD, but that inhibition was prevented by semaglutide [63]; and addition of GPL-1 itself to cultured endothelial cells also inhibited autophagy, in this instance associated with reduced ROS [30]. Semaglutide has promoted benefit to cognition in animal studies [64,65,66], but as mentioned above, those may be inapplicable to the human situation. It is notable that there are no reports showing benefits to cognition in humans from using semaglutide. That lack is surprising because there are two large trials using the drug in AD.

### 4.6. Dulaglutide and Cognition or Dementia

There is only one study of dulaglutide and cognition in humans, but it was a very large study and produced convincing results. Those results are possibly due to dulaglutide’s excellent uptake in brain, which was 61.8%, compared with which the brain uptake was only 28% for exenatide, 14% for lixisinatide, and virtually zero for liraglutide, semaglutide, and tirzepatide [44]. That very large study of dulaglutide was a randomized, double-blind placebo-controlled trial of subjects aged ≥50 years, with either established or newly diagnosed type 2 diabetes and additional cardiovascular risk factors; cognitive function was assessed at baseline and during follow-up using the Montreal Cognitive Assessment (MoCA) and Digit Symbol Substitution Test (DSST) [67]. During a median follow-up of 5.4 years, 8828 participants provided a baseline and one or more follow-up MoCA or DSST scores, of whom 4456 had been assigned dulaglutide and 4372 assigned placebo. The cognitive outcome was the first occurrence of a follow-up score in the MoCA or DSST that was ≥1.5 standard deviations below the baseline mean score in the participants’ country; that occurred in 4.05/100 patient-years in those assigned dulaglutide and in 4.35/100 patient years in those assigned placebo. After adjustment for individual, standardized baseline scores, the hazard of substantive cognitive impairment was reduced by 14% in those assigned dulaglutide (HR 0.86, *p* = 0.0018). The mechanism for this benefit may be because dulaglutide reduced the disadvantageous, hyperphosphorylation of tau and neurofibrillary tangles, via improving the PI3K/AKT/GSK3β signaling pathway [68].

The author is unaware of any other data in humans that support or deny a benefit to cognition from the use of dulaglutide. However, the study described in the above paragraph contained 8828 participants; that large number makes it unusually robust and its results provide a substantial likelihood, admittedly not certainty, that dulaglutide may benefit cognition in humans. Other future studies in humans that involve large numbers of participants would be a welcome addition to the knowledge base. In brief, human trials assessing cognition, using commercially available GLP-1 agonists, showed significant benefit from dulaglutide, variable results from very small trials using liraglutide and exenatide, and no report of benefit from semaglutide. The data support a hypothesis that dulaglutide might cure AD.

A clinical trial is required in order to test the hypothesis that dulaglutide cures AD to a degree that is 30%, beyond that provided by an equipoise treatment with lecanemab plus memantine, as well as to test the validity and safety of the suggested therapy. It is important to note that if, during the three months before or after the diagnosis of AD, known risk factors either appeared for the first time or, if already present, clearly worsened, they should be treated with standard therapy in addition to the dulaglutide or equipoise treatment.

## 5. Conclusions and Summary

- GLP-1 agonist drugs address the dysfunctions of all brain cell types in Alzheimer’s disease and therefore may cure the dementia.
- An additional benefit of GLP-1 agonist drugs is their reversal of ROS and its cytotoxicity.
- Among the available GLP-1 agonists, dulaglutide might be more effective for curing AD than are others.
- A clinical trial would have, as its primary outcome, a 30% greater cure rate of AD than achieved by an equipoise arm of lithium plus memantine.

## Data Availability

For data, please see relevant citations.
